# Copy number increase of *ACTN4* is a prognostic indicator in salivary gland carcinoma

**DOI:** 10.1002/cam4.214

**Published:** 2014-02-27

**Authors:** Yukio Watabe, Taisuke Mori, Seiichi Yoshimoto, Takeshi Nomura, Takahiko Shibahara, Tesshi Yamada, Kazufumi Honda

**Affiliations:** 1 Division of Chemotherapy and Clinical Research, National Cancer Center Research InstituteTokyo, 104-0045, Japan; 2 Department of Oral and Maxillofacial Surgery, Tokyo Dental CollegeChiba, 261-8502, Japan; 3 Division of Molecular Pathology, National Cancer Center Research InstituteTokyo, 104-0045, Japan; 4 Department of Head and Neck Oncology, National Cancer Center HospitalTokyo, 104-0045, Japan

**Keywords:** Actinin-4, *ACTN4*, head and neck cancer, prognostic marker, salivary gland carcinoma

## Abstract

Copy number increase (CNI) of *ACTN4* has been associated with poor prognosis and metastatic phenotypes in various human carcinomas. To identify a novel prognostic factor for salivary gland carcinoma, we investigated the copy number of *ACTN4*. We evaluated DNA copy number of *ACTN4* in 58 patients with salivary gland carcinoma by using fluorescent in situ hybridization (FISH). CNI of *ACTN4* was recognized in 14 of 58 patients (24.1%) with salivary gland carcinoma. The cases with CNI of *ACTN4* were closely associated with histological grade (*P* = 0.047) and vascular invasion (*P* = 0.033). The patients with CNI of *ACTN4* had a significantly worse prognosis than the patients with normal copy number of *ACTN4* (*P* = 0.0005 log-rank test). Univariate analysis by the Cox proportional hazards model showed that histological grade, vascular invasion, and CNI of *ACTN4* were independent risk factors for cancer death. Vascular invasion (hazard ratio [HR]: 7.46; 95% confidence interval [CI]: 1.98–28.06) and CNI of *ACTN4* (HR: 3.23; 95% CI: 1.08–9.68) remained as risk factors for cancer death in multivariate analysis. Thus, CNI of *ACTN4* is a novel indicator for an unfavorable outcome in patients with salivary gland carcinoma.

## Introduction

Salivary gland carcinomas are rare malignant tumors comprising about 5% of cancers of the head and neck region 1. In addition, the histopathology of salivary gland tumors is diverse. The classification system of the World Health Organization (WHO) contains at least 24 histopathological types of salivary gland carcinomas. The management of salivary gland carcinomas can be confusing due to the extreme diversity of tumor types, their relative rarity, the requirement for long-term follow-up, and strategy for treatment in many instances to predict outcome. Although the clinical parameters, such as clinical stage, age, and tumor location, are important for the prognostic factors in salivary gland carcinoma, histological grading also ranks highly as a critical prognostic factor. Histological grading may stratify the risk of lymph node metastasis and give a rationale for the extent of surgery and the need for adjuvant therapy 2. If surrogate biomarkers associated with histological grade and/or outcome could be identified, they would become powerful indicators of the optimal treatment strategy for patients with salivary gland carcinoma.

We identified actinin-4, an actin-bundling protein encoded by *ACTN4*, as a biomarker that could be used to evaluate the invasion and metastasis capabilities of cancer cells 3. The overexpression of actinin-4 proteins was closely associated with the invasive phenotypes of some cancers, such as breast 3, colorectal 4,5, ovarian 6,7, bladder 8,9, oral squamous cell 10, pancreas 11,12, and lung carcinomas 13–15. Oncogene amplification is often observed in aggressive malignant phenotypes of cancer 16. Recently, it has been reported that the amplification of *ACTN4* can strictly predict the clinical outcome, and *ACTN4* has been recognized as an oncogene 6,11,13.

In the present study, we investigated the copy number of *ACTN4* in salivary gland carcinomas by using fluorescent in situ hybridization (FISH). Copy number increase (CNI) of *ACTN4* was positively associated with histological grade and poor outcome. We identified the biomarker to predict the outcome of salivary gland carcinoma.

This is the first report to examine the clinical usage of CNI of *ACTN4* as a prognostic factor in salivary gland carcinoma.

## Patients and Methods

### Patients and tissue samples

We reviewed the clinicopathological records of 58 patients who underwent surgical resection with curative intention for salivary gland carcinoma at the National Cancer Center Central Hospital (Tokyo, Japan) between 1997 and 2011.

Formalin-fixed paraffin-embedded tissue samples of 58 salivary gland carcinomas and 10 normal submandibular gland or parotid gland specimens were collected and reviewed in our institution (T. M.) according to the WHO classification of salivary gland carcinomas (Table 1). Histological grade was determined according to the three-tiered grading system proposed by Jouzdani 17.

**Table 1:** Association of *ACTN4* with clinicopathological characteristics of salivary gland cancer patients

	*ACTN4* FISH			Actinin-4 IHC		
				
	NCN	CNI	*P*-value	Negative (0, +1)	Positive (+2, +3)	*P*-value
Total	44 (75.9%)	14 (24.1%)		19 (32.8%)	39 (67.2%)	
ADCC	20 (95.2%)	1 (4.8%)		3 (14.3%)	18 (85.7%)	
CAEPA	8 (72.7%)	3 (27.3%)		5 (45.5%)	6 (54.5%)	
EMYC	2 (66.7%)	1 (33.3%)		0	3 (100%)	
MYC	0	1 (100%)		1 (100%)	0	
ACCC	3 (100%)	0		1 (33.3%)	2 (66.7%)	
ACNOS	5 (71.4%)	2 (28.6%)		4 (57.1%)	3 (42.9%)	
MEC	3 (75.0%)	1 (25.0%)		2 (50.0%)	2 (50.0%)	
SDC	2 (50.0%)	2 (50.0%)		2 (50.0%)	2 (50.0%)	
SC	1 (33.3%)	2 (66.7%)		0	3 (100%)	
OC	0	1 (100%)		1 (100%)	0	
Age						
<67 years	26 (83.9%)	5 (16.1%)	0.1267	9 (29.0%)	22 (71.0%)	0.5170
≥67 years	18 (66.7%)	9 (33.3%)		10 (37.0%)	17 (63.0%)	
Gender						
Men	24 (75.0%)	8 (25.0%)	0.8648	13 (40.6%)	19 (59.4%)	0.1567
Women	20 (76.9%)	6 (23.1%)		6 (23.1%)	20 (76.9%)	
Size						
T1–T2	12 (100%)	0	0.0503	4 (33.3%)	8 (66.7%)	1.000
T3–T4	28 (68.3%)	13 (31.7%)		14 (34.1.%)	27 (65.9%)	
Unknown	4 (80.0%)	1 (20.0%)		1 (20.0%)	4 (80.0%)	
Lymph node metastasis						
Absent	31 (79.5%)	8 (20.5%)	0.5141	10 (25.6%)	29 (74.4%)	0.0980
Present	13 (68.4%)	6 (31.6%)		9 (47.4%)	10 (52.6%)	
Histological grade						
Low, intermediate	**26 (86.7%)**	**4 (13.3%)**	0.0465*	7 (23.3%)	23 (76.7%)	0.1134
High	**18 (64.3%)**	**10 (35.7%)**		12 (42.9%)	16 (57.1%)	
Neural invasion						
Absent	23 (76.7%)	7 (23.3%)	0.8822	11 (36.7%)	19 (63.3%)	0.5116
Present	21 (75.0%)	7 (25.0%)		8 (28.6%)	20 (71.4%)	
Vascular invasion						
Absent	**36 (83.7%)**	**7 (16.3%)**	0.0326*	13 (29.5%)	31 (70.5%)	0.5141
Present	**8 (53.3%)**	**7 (46.7%)**		6 (42.9%)	8 (57.1%)	

FISH, fluorescent in situ hybridization; IHC, immunohistochemistry; ADCC, adenoid cystic carcinoma; CAEPA, carcinoma ex pleomorphic adenoma; EMYC, epithelial-myoepithelial carcinoma; MYC, myoepithelial carcinoma; ACCC, acinic cell carcinoma; ACNOS, adenocarcinoma, not otherwise specified; MEC, mucoepidermoid carcinoma; SDC, salivary duct carcinoma; SC, sebaceous carcinoma; OC, oncocytic carcinoma; NCN, normal copy number; CNI, copy number increase.

* *P* < 0.05. Statistically significant associations are highlighted in bold.

This study was approved by the ethics committee of the National Cancer Center (approval #2010-0759).

### TMA construction

Tissue microarrays (TMAs) were prepared from formalin-fixed paraffin-embedded pathological blocks, as previously described 18. TMA blocks were cut into 4-*μ*m-thick sections and subjected to FISH and immunohistochemistry (IHC) 4,11.

### Fluorescence in situ hybridization

The FISH probe of bacterial artificial chromosome clone containing *ACTN4* and chromosome 19p (a control clone) was purchased from Abnova (Taipei, Taiwan) 13.The labeled bacterial artificial chromosome clone DNA was subjected to FISH as previously described. TMAs were hybridized with FISH probes at 37°C for 48 h. The nuclei were counterstained with 4, 6-duamidino-2-phenylindone. The number of fluorescence signals corresponding to the copy number of *ACTN4* and control signals in the nuclei of 20 interphase tumor cells was counted (Y. W. and K. H.).

FISH patterns were defined as described previously 19,20. Briefly, the samples were grouped as normal disomy (two or less *ACTN4* signals in more than 90% of cells), low polysomy (four or more *ACTN4* signals in more than 10% but less than 40% of tumor cells), high polysomy (four or more *ACTN4* signals in more than 40% of tumor cells), and gene amplification (ratio *ACTN4*/chromosome more than 2, or 15 copies in more than 10% of tumor cells) 19,20.

### Immunohistochemistry

The anti-actinin-4 monoclonal antibody (13G9), which we originally established, was purchased from Transgenic Inc. (Kumamoto, Japan) 14. Immunostaining of actinin-4 proteins was performed with the Ventana DABMap detection kit and an automated slide stainer (Discovery XT; Ventana Medical System, Tucson, AZ) 13,14. The expression level of actinin-4 protein was classified as: no expression (immunoreactivity score, 0), in which no tumor cells were stained with anti-actinin-4 antibody; weak expression (+1), in which tumor cells were stained with weaker intensity than endothelial cells; moderate expression (+2), in which less than 30% of tumor cells were stained; and strong expression (+3), in which more than 30% of tumor cells were stained. Two independent investigators (Y. W. and T. M.) who had no clinical information about the cases evaluated the staining patterns.

### Statistical analysis

Significant differences were detected by using the Mann–Whitney *U* test, Student's *t*-test, Pearson's chi-square test, and Fisher's exact test. Overall survival was measured as the period from surgery to the date of death or last follow-up and was estimated by the Kaplan–Meier analysis. Differences between the overall survival curves were assessed with the log-rank test. Univariate and multivariate analyses were performed with the Cox regression model. Data were analyzed with the StatFlex statistical software package (version 6.0; Artiteck, Osaka, Japan) or the R-project (http://www.r-project.org/) 11,13,14.

## Results

### Determination of the copy number of *ACTN4* by FISH

**Figure 1:**
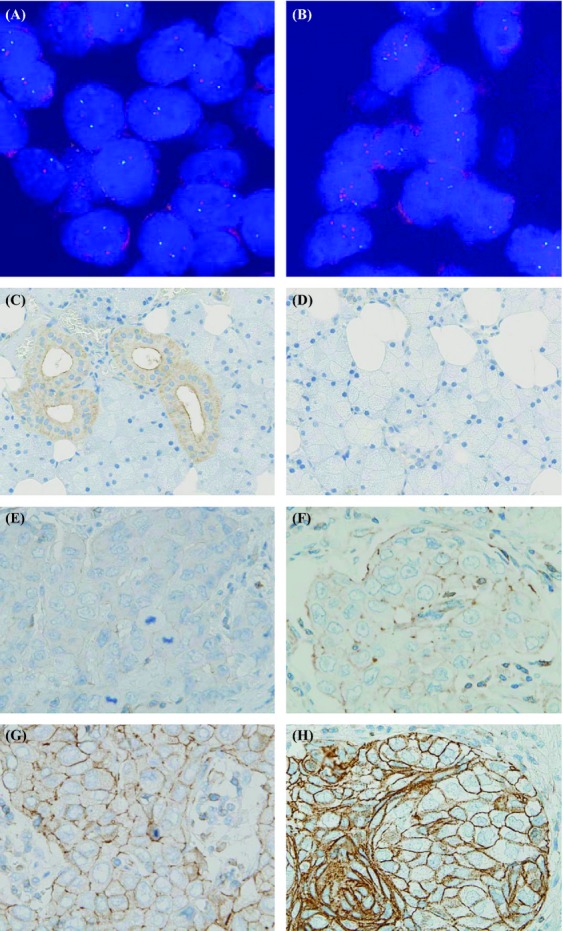
Representative copy number status of *ACTN4* in salivary gland cancer determined by fluorescence in situ hybridization (FISH) (A and B). Representative expression of actinin-4 protein in normal salivary gland (C and D) and salivary gland cancer (E–H), as determined by immunohistochemistry (IHC). (A) Disomy of *ACTN4* in an adenoid cystic carcinoma (ADCC), (B) gene amplification of *ACTN4* in ADCC. (C) striated duct, (D) acinar gland, (E) no expression of actinin-4 protein in mucoepidermoid carcinoma (immunoreactivities score 0), (F) weak expression, in salivary duct carcinoma (+1), (G) moderate expression in salivary duct carcinoma (+2), (H) strong expression in sebaceous carcinoma (+3).

We determined the copy number of *ACTN4* in salivary gland carcinomas by using FISH. Among the 58 tumors, 33 exhibited normal disomy (56.9%), 11 exhibited low polysomy (19.0%), 10 exhibited high polysomy (17.2%), and four exhibited gene amplification (6.9%) (Table 1). Tumors with normal disomy and low polysomy were defined as having normal copy number (NCN) of *ACTN4*, and tumors with high polysomy and gene amplification were defined as having a CNI of *ACTN4*, according to the definition of FISH analysis for epidermal growth factor receptor 1 (*EGFR*) (Fig. 1A and B). Fourteen of 58 tumors (24.1%) exhibited CNI, and 44 of 58 tumors exhibited NCN (75.9%). Histologically, the CNI was recognized in adenoid cystic carcinoma (ADCC) (4.8%, 1/21), carcinoma ex pleomorphic adenoma (CAEPA) (3/11, 27.3%), epithelial-myoepithelial carcinoma (EMYC) (1/3, 33.3%), myoepithelial carcinoma (MYC) (1/1, 100%), adenocarcinoma not otherwise specified (ACNOS) (2/7, 28.6%), mucoepidermoid carcinoma (MEC) (1/4, 25.0%), salivary duct carcinoma (SDC) (2/4, 50%), sebaceous carcinoma (SC) (2/3, 66.7%) and oncocytic carcinoma (OC) (1/1 100%). The NCN and CNI groups had statistically significant differences in histological grade (*P* = 0.0465) and vascular invasion (*P* = 0.0326); however, there were no statistically significant differences in histology, age, gender, size, lymph node metastasis, or neural invasion (Table 1).

### Protein expression of actinin-4 determined by IHC

We investigated the protein expression level of actinin-4 by using IHC. Among the 58 tumors, 14 (24.1%) had strong (+3) expression, 25 (43.1%) had moderate (+2) expression, 13 (22.4%) had weak (+1) expression, and six (10.3%) had no (0) expression.

Positive staining for actinin-4 protein, which was defined as moderate expression (+2) and strong expression (+3), occurred in 39 of 58 tumors (67.2%) (Fig. 1E–H). The distribution of actinin-4 protein in histological subtypes is described in Table 1. There were no statistically significant differences between the positive and negative staining groups in terms of age, gender, size, lymph node metastasis, histological grade, neural invasion, or vascular invasion (Table 1).

In normal submandibular salivary glands, actinin-4 proteins were equally expressed in acinar cells, intercalated duct cells, striated duct cells and endothelial cells. In contrast, in the parotid gland, the protein expression level of actinin-4 in acinar cells was weaker than in ductal cells (Fig. 1C and D).

### The correlation between copy number of *ACTN4* and protein expression of actinin-4

**Figure 2:**
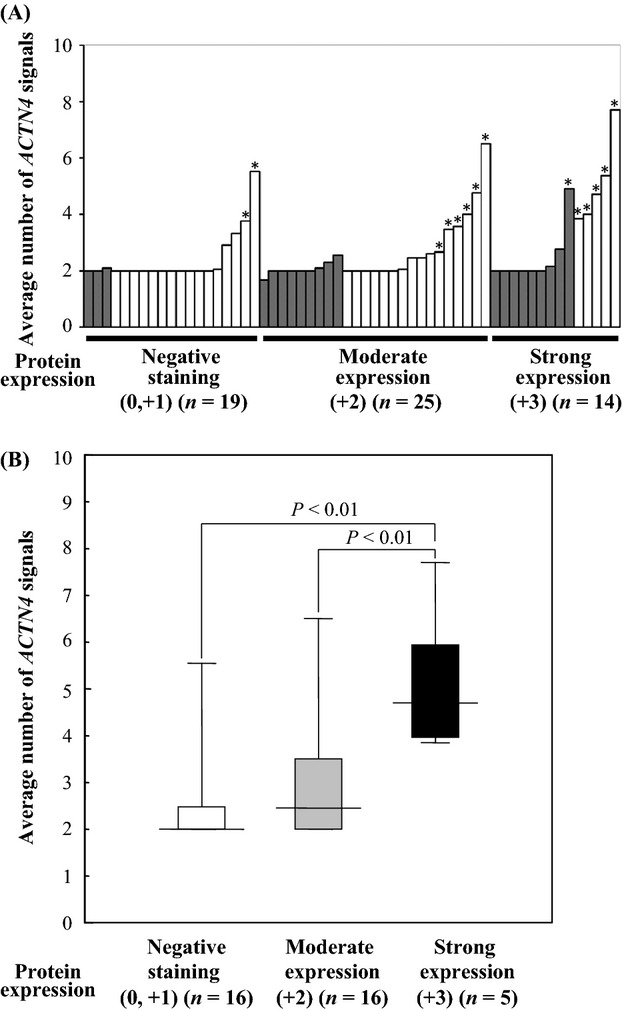
Collation between copy numbers of *ACTN4* and actinin-4 protein. (A) Bar graph of copy numbers of *ACTN4* in individual patients (*y*-axis; average number of *ACTN4* signals, gray bars; patients with adenoid cystic carcinoma (ADCC), white bars; patients with salivary gland carcinomas excluding ADCC, *; the patients with copy number increase [CNI]). (B) Box and whisker plot for average number of *ACTN4* signals between protein expression levels of actinin-4 proteins in the patients with salivary gland carcinomas excluding ADCC. The average number of *ACTN4* signals in the patients with strong expression of actinin-4 protein was significantly higher than the patients with moderate expression or negative staining of actinin-4 protein (*P* < 0.01, Student's *t*-test).

We confirmed the correlation between *ACTN4* copy number and protein expression of actinin-4. CNI of *ACTN4* was recognized in 12 of 39 (30.8%) tumors with positive staining of actinin-4. CNI was recognized in six of 14 (42.9%) tumors with strong (+3) expression of actinin-4, six of 25 (24.0%) tumors with moderate (+2) expression, and two of 19 (10.5%) tumors with negative (0 and +1) staining (Fig. 2A). Although 18 of 21 ADCCs were positively stained for actinin-4 (85.7%), CNI of *ACTN4* was recognized in only one ADCC (4.8%) (Fig. 2A). Therefore, we considered that the expression level of actinin-4 protein is not positively associated with CNI of *ACTN4* in ADCC. We then investigated the correlation between protein expression levels and copy number of *ACTN4* in salivary gland carcinomas excluding ADCCs. The average copy numbers of *ACTN4* in salivary gland carcinomas excluding ADCC were 5.12, 2.90, and 2.47 in tumors with strong expression (+3), moderate expression (+2), and negative staining (0 and +1), respectively. Copy numbers of *ACTN4* were significantly increased in tumors with strong expression (+3) of actinin-4 in comparison with negative staining (0 and +1) and moderate expression (+2) (*P* < 0.01) in salivary gland carcinomas, excluding ADCC (Fig. 2B).

### The prognostic significance of CNI of *ACTN4*

**Figure 3:**
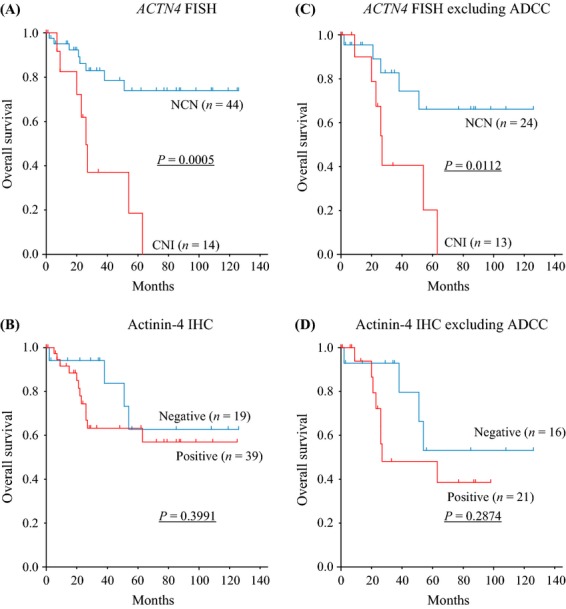
Overall survival curves of patients with salivary gland carcinomas, including adenoid cystic carcinoma (ADCC) (A and B) or excluding ADCC (C and D), by evaluations of fluorescence in situ hybridization (FISH) (A and C) or immunohistochemistry (IHC) (B and D). The statistical significances were recognized in evaluation of FISH between copy number increase (CNI) and normal copy number (NCN) in patients with salivary gland carcinomas including/excluding ADCC (A and C). In contrast, the statistical significance was not recognized in an evaluation of IHC in both cohorts (B and D).

Kaplan–Meier analysis revealed that CNI of *ACTN4* was significantly correlated with poor outcome in overall survival of the 58 patients with salivary gland carcinoma, including ADCC (*P* = 0.0005, log-rank test) (Fig. 3A). ADCC has a better prognosis than high-grade histological subtypes of salivary gland carcinoma 21. The correlation analysis between CNI and protein expression in ADCC (Fig. 2A, gray bars) revealed that although 85.7% of ADCCs had strong expression (+3) or moderate expression (+2) of actinin-4 protein, CNI of *ACTN4* was recognized in only one tumor. To remove the bias of the unique prognosis of ADCC, we also investigated the prognostic significance with CNI of *ACTN4* in 37 salivary gland carcinoma patients excluding ADCC. A statistically significant difference in prognosis was recognized between patients with NCN and patients with CNI (*P* = 0.0112); the overall survival of patients with CNI was worse than patients with NCN (Fig. 3C). In contrast, the actinin-4 protein expression level was not statistically correlated to overall survival in salivary gland carcinomas when including or excluding ADCC (Fig. 3B and D).

### HR for death in patients with salivary gland carcinoma

We calculated the hazard ratios (HR) of some parameters, including age, gender, size, lymph node metastasis, histological grade, neural invasion, vascular invasion, actinin-4 protein expression, and CNI of *ACTN4*, for death by using univariate and multivariate Cox regression analysis.

In the patients with salivary gland carcinomas including ADCC, histological grade (HR: 4.69; 95% confidence interval [CI]: 1.50–14.61), vascular invasion (HR: 10.86; 95% CI: 3.56–33.14), and CNI of *ACTN4* (HR: 5.21; 95% CI: 1.92–14.19) remained as positive predictors by using univariate analysis, and multivariate analysis revealed that vascular invasion (HR: 7.46; 95% CI: 1.98–28.06) and CNI of *ACTN4* (HR: 3.23; 95% CI: 1.08–9.68) were independent positive predictors for death in patients with salivary gland carcinoma (Table 2).

**Table 2:** Hazard ratios for death in salivary gland cancer patients

	Univariate analysis^1^			Multivariate analysis^1^		
		
Variable	HR	95% CI	*P*-value	HR	95% CI	*P*-value
Age						
<67/≥67 years	2.69	0.93–7.78	0.067			
Gender						
Women/men	1.20	0.45–3.24	0.714			
T classification						
T1–T2/T3–T4	2.28	0.51–10.11	0.279			
Lymph node metastasis						
Absent/present	2.51	0.93–6.75	0.069			
Histological grade						
Low, intermediate/high	4.69	1.50–14.61	**0.007765**	1.32	0.31–5.45	0.701222
Neural invasion						
Absent/present	1.38	0.51–3.71	0.524			
Vascular invasion						
Absent/present	10.86	3.56–33.14	**0.000028**	7.46	1.98–28.06	**0.002958**
Actinin-4 IHC						
Negative/positive	1.64	0.53–5.10	0.394			
*ACTN4* FISH						
NCN/CNI	5.21	1.92–14.19	**0.001230**	3.23	1.08–9.68	**0.035815**

HR, hazard ratio; CI, confidence interval; FISH, fluorescent in situ hybridization; IHC, immunohistochemistry; NCN, normal copy number; CNI, copy number increase.

1 Univariate and multivariate analysis with Cox proportional hazards regression model. *P*-values of <0.05 are shown in bold.

We also calculated the HRs for death in patients with salivary gland carcinomas excluding ADCC. Univariate analysis revealed that vascular invasion (HR: 8.58; 95% CI: 2.16–34.11) and CNI of *ACTN4* (HR: 4.18; 95% CI: 1.29–13.53) were significant positive predictors for death in salivary gland carcinomas excluding ADCC. Multivariate analysis also revealed that vascular invasion and CNI of *ACTN4* were independent risk factors for both salivary gland carcinomas including and excluding ADCC (Table 3).

**Table 3:** Hazard ratios for death in salivary gland cancer patients excluding ADCC

	Univariate analysis^1^			Multivariate analysis^1^		
		
Variable	HR	95% CI	*P*-value	HR	95% CI	*P*-value
Age						
<67/≥67 years	2.52	0.68–9.41	0.1688			
Gender						
Women/men	2.10	0.57–7.82	0.2670			
T classification						
T1–T2/T3–T4	1.68	0.36–7.81	0.5075			
Lymph node metastasis						
Absent/present	1.55	0.50–4.83	0.4483			
Histological grade						
Low, intermediate/high	2.81	0.76–10.44	0.1228			
Neural invasion						
Absent/present	1.50	0.47–4.72	0.4932			
Vascular invasion						
Absent/present	8.58	2.16–34.11	**0.0023**	9.00	2.15–37.61	**0.0026**
Actinin-4 IHC						
Negative/positive	1.91	0.57–6.43	0.2912			
*ACTN4* FISH						
NCN/CNI	4.18	1.29–13.53	**0.0168**	4.35	1.28–14.87	**0.0187**

ADCC, adenoid cystic carcinoma; HR, hazard ratio; CI, confidence interval; FISH, fluorescent in situ hybridization; IHC, immunohistochemistry; NCN, normal copy number; CNI, copy number increase.

1 Univariate and multivariate analysis with Cox proportional hazards regression model. *P*-values of <0.05 are shown in bold.

## Discussion

The assessment of prognostic factors in salivary gland carcinoma is difficult due to its low frequency and morphological diversity 22. Histological grading of salivary gland carcinoma is an important predictor of survival 23. It can stratify the risk of lymph node metastases and provide information for deciding the treatment strategy, including the extent of surgery and the use of adjuvant radiotherapy 24. In the present study, we identified a novel predictor for the prognosis of salivary gland carcinoma and found that it was significantly associated with histological grade.

Our laboratory identified the *ACTN4* gene product as an actin-bundling protein that was closely associated with cell movement and cancer invasion 3. In a previous study, colorectal cancer cell lines in which actinin-4 was overexpressed stimulated invasive cellular protrusions and had a significantly more invasive phenotype than control cells 4. Moreover, pancreatic and oral squamous cell carcinoma cells in which actinin-4 expression was reduced with siRNA exhibited decreased invasiveness 10,11. An orthotopic transplantation study of cells overexpressing actinin-4 revealed that the regional lymphatic metastasis and destructive invasion to stromal cells were significantly increased in colorectal 4 and pancreatic cancer 11.

Actinin-4 overexpression was also confirmed in solid malignant tumors that had been surgically excised, and protein expression was an unfavorable predictor of patient outcome. One cause of actinin-4 protein overexpression is *ACTN4* gene amplification. In fact, gene amplification of *ACTN4* has been detected in tumors from patients with pancreatic 11, ovarian 6,7, and lung cancers 13, and correlations between protein expression and gene amplification have been statistically recognized in some cancers. Especially, Noro et al. reported that *ACTN4* amplification could more strictly predict poor outcome than actinin-4 protein expression in stage-I adenocarcinoma of the lung 13. To identify the specificity of gene amplification of *ACTN4*, we previously examined the correlation of gene amplification of a gene near *ACTN4*. V-akt murine thymoma viral oncogene homolog 2 (*AKT2*) is located on 19q13, and it is near *ACTN4*. The distance between *ACTN4* and *AKT2* is 1.6 Mbp. We previously reported that coamplification of *AKT2* and *ACTN4* did not necessarily accord for invasion of pancreatic cancer 5,11. The present study is the first report that CNI of *ACTN4*, including gene amplification and high polysomy of *ACTN4*, was significantly correlated to histological grade and vascular invasion in salivary gland carcinoma. CNI of *ACTN4* was recognized at a frequency greater than 20% in OC (1/1, 100%), MYC (1/1, 100%), SC (2/3, 66.7%), SDC (2/4, 50.0%), EMYC (1/3, 33.3%), ACNOS (2/7, 28.6%), CAEPA (3/11, 27.3%), and MEC (1/4, 25.0%). However, CNI of *ACTN4* was not found in patients with acinic cell carcinoma (ACCC) (0/3, 0%) and was rarely observed in patients with ADCC (1/21, 4.8%). The survival time of the patient with CNI of *ACTN4* in ADCC was 7 months from the first treatment. Despite the rare frequency of CNI of *ACTN4* in ADCC, overexpression of actinin-4 protein was recognized in 85.7% of patients with ADCC. ADCC has several cellular components constructed by ductal epithelial, myoepithelial, and basement cells, and protein expression of actinin-4 is particularly recognized in myoepithelial cells of normal salivary glands. Therefore, it was considered that the protein expression of actinin-4 was dependent on the histological phenotype; however, this was not associated with the genetic alteration that was dependent on malignant change.

-ADCC was different from other subtypes of salivary gland carcinomas, we investigated the correlation between CNI and protein expression of actinin-4 by using 37 patients with salivary gland carcinomas excluding ADCC. Significant correlations were recognized between increased copy numbers of *ACTN4* and protein expression levels of actinin-4. This data suggests that the overexpression of actinin-4 protein was stimulated by CNI of *ACTN4*. Cox regression univariate analysis revealed that histological grade, vascular invasion, and CNI of *ACTN4* were risk factors for cancer death in salivary gland carcinoma patients with or without ADCC. The HR of CNI of *ACTN4* for death was higher than the HR for the histological grade. In addition, although multivariate analysis revealed that CNI of *ACTN4* and vascular invasion were independent risk factors for tumor death, histological grade did not remain as an independent risk factor. These results suggest that CNI of *ACTN4* may have a greater impact than histological grade on patient death. Although CNI of *ACTN4* was significantly correlated with histological grade and vascular invasion, the protein expression of actinin-4 was not associated with any clinical factors in salivary gland carcinoma. In addition, protein expression could not predict an unfavorable outcome in patients with salivary gland carcinoma. Although CNI of *ACTN4* was dominantly recognized in salivary gland carcinoma with invasive phenotypes, it was recognized in only one of 21 cases with ADCC; therefore, we considered the possibility that, due to the overexpression of actinin-4 that was frequently observed in ADCC patients, protein expression is not correlated to an unfavorable prognosis in salivary gland carcinoma. In contrast, protein expression of actinin-4 was recognized in 18 cases with ADCC. An explanation for this observation involved the discrepancy between CNI and protein expression of actinin-4 in ADCC. We analyzed the correlation between overall survival and protein expression of actinin-4 in patients excluding ADCC; although statistical significance was not recognized, it seemed that overall survival in the positive staining group had a poorer prognosis than in the negative staining group. To probe the statistical significance of protein expression of actinin-4, we considered that the power of statistical hypothesis testing was not enough. More non-ADCC salivary gland carcinoma samples are needed to prove a significant correlation between overall survival and protein expression. Moreover, although CNI can quantitatively evaluate the copy numbers of the *ACTN4* gene, our evaluation system for protein expression of actinin-4 cannot quantitatively classify the cases with protein expression of actinin-4. Therefore, we considered that CNI of *ACTN4* more strictly predicted the vascular invasion of cancer cells and poor prognosis than protein expression of actinin-4. Similar observations have also been recognized in ovarian cancer. We previously reported that the gene amplification of *ACTN4* could predict the prognosis of patients with advanced stage ovarian cancer more accurately than protein expression of actinin-4 6.

Ettl et al. reported the occurrence of genomic aberrations of the tyrosine kinase receptors *EGFR*, human epidermal growth factor receptor 2 (*HER2*), and hepatocyte growth factor (*MET*) as well as phosphatase and tensin homolog on chromosome 10 (*PTEN*) in different subtypes of salivary gland carcinomas 25,26, which have a strong impact on overall survival 27. In addition, they also reported that the metastasis of cervical lymph nodes also correlated with copy number gain of *EGFR* and *HER2*, aberration of *MET*, and *PTEN* 20. Moreover some translocation and fusion genes are found frequently in MEC t(11;19) (CRYC1-MAML2) 28 or ADCC t(6;9) (MYB-NFIB) 29 and have a prognostic impact. These genetic alterations are considered as a driver for malignant phenotype, and molecular-targeted therapy has gained attention as a new therapeutic strategy for salivary gland carcinomas. In fact, clinical trials with some inhibitors or antibodies for molecular targets, such as gefitinib (a small-molecule *EGFR* inhibitor), cetuximab (an anti-EGFR antibody), and trastuzumab (an anti-HER2 antibody), were performed for patients with salivary gland carcinomas. Although the results of phase II clinical trials of gefitinib, cetuximab, and trastuzumab have been reported 30,31, standard molecular-targeted therapy has not yet been established for salivary gland carcinoma.

*ACTN4* is located on chromosome 19q13.1 11. Genetic alterations of 19q13.1 and *ACTN4* have not yet been reported in salivary gland carcinoma. Although a large-scale prospective study to prove the clinical significance for *ACTN4* is necessary, we conclude that *ACTN4* is a surrogate biomarker for predicting prognosis to support histological grading in salivary gland carcinoma and that the inhibition of the biological function of *ACTN4* may impact a new therapeutic strategy for high-grade salivary gland carcinoma.
